# Supplementary material for: Machine Learning Accelerates Crystallization for Structure Determination
Source: Angew Chem Int Ed Engl. 2026 May 4;65(25):e1218503. doi: 10.1002/anie.1218503 (PMC13266958; doi:10.1002/anie.1218503)

# checkCIF/PLATON report

Structure factors have been supplied for datablock(s) lcz111

THIS REPORT IS FOR GUIDANCE ONLY. IF USED AS PART OF A REVIEW PROCEDURE FOR PUBLICATION, IT SHOULD NOT REPLACE THE EXPERTISE OF AN EXPERIENCED CRYSTALLOGRAPHIC REFEREE.

No syntax errors found. CIF dictionary Interpreting this report

# Datablock: lcz111

---

```
Bond precision:   C-C = 0.0130 A          Wavelength=1.54184

Cell:            a=10.6616(2)     b=12.9514(2)        c=24.6669(4)
                 alpha=90         beta=102.471(2)     gamma=90
Temperature:     100 K

                Calculated                  Reported
Volume          3325.71(10)                 3325.71(10)
Space group     P 21                        P 1 21 1
Hall group      P 2yb                       P 2yb
                                            C52 H70 Ag3 F18 N7 O13, H2
Moiety formula  C52 H72 Ag3 F18 N7 O14      O
Sum formula     C52 H72 Ag3 F18 N7 O14      C52 H72 Ag3 F18 N7 O14
Mr              1684.78                     1684.77
Dx,g cm-3       1.683                       1.682
Z               2                           2
Mu (mm-1)       8.044                       8.044
F000            1696.0                      1696.0
F000'           1703.14
h,k,lmax        13,16,31                    13,16,30
Nref            14314[ 7484]                12106
Tmin,Tmax       0.317,0.324                 0.640,1.000
Tmin'           0.240

Correction method= # Reported T Limits: Tmin=0.640 Tmax=1.000
AbsCorr = MULTI-SCAN
```

```
Data completeness= 1.62/0.85        Theta(max)= 78.251

                                                  wR2(reflections)=
R(reflections)= 0.0423( 11032)                    0.1193( 12106)

S = 1.104                Npar= 866
```

---

The following ALERTS were generated. Each ALERT has the format
**test-name_ALERT_alert-type_alert-level**.
Click on the hyperlinks for more details of the test.

---

### Alert level B

```
PLAT420_ALERT_2_B D-H Bond Without Acceptor  O1       --H1B      .     Please Check
```

**Author Response: The solvent molecules are highly disordered, which supposed to form hydrogen bond with O have been removed by the SQUEEZE routine in the PLATON software package.**

---

### Alert level C

```
PLAT042_ALERT_1_C Calc. and Reported MoietyFormula Strings  Differ     Please Check
              Calc: C52 H72 Ag3 F18 N7 O14
              Rep.: C52 H70 Ag3 F18 N7 O13, H2 O
PLAT213_ALERT_2_C Atom F02D          has ADP max/min Ratio .....        3.3 prolat
PLAT234_ALERT_4_C Large Hirshfeld Difference F018     --C01Y     .       0.17 Ang.
PLAT234_ALERT_4_C Large Hirshfeld Difference F02I     --C02C     .       0.16 Ang.
PLAT241_ALERT_2_C High   'MainMol' Ueq as Compared to Neighbors of        O1 Check
PLAT342_ALERT_3_C Low Bond Precision on  C-C Bonds ..............      0.013 Ang.
PLAT911_ALERT_3_C Missing FCF Refl Between Thmin & STh/L=    0.600        22 Report
               12  0  0,   4 10  0,  12  0  1,   5  5  1,  12  0  2,  12  1  2,
               10  7  2,  12  0  3,  12  1  3,  10  7  3,  12  0  4,   2  7  4,
               10  7  4,  10  6  5,  10  7  5,  -6  0  6,  -6  1  6,  -6  0  7,
               -6  1  7,  10  6  9,  -1  7 25,  -1  8 25,
PLAT915_ALERT_3_C No Flack x Check Done: Low Friedel Pair Coverage         71 %
```

---

### Alert level G

```
PLAT007_ALERT_5_G Number of Unrefined Donor-H Atoms .............          7 Report
              H00C  H1A   H1B   H00E  H00G  H00I  H00O
PLAT242_ALERT_2_G Low    'MainMol' Ueq as Compared to Neighbors of       C01B Check
PLAT242_ALERT_2_G Low    'MainMol' Ueq as Compared to Neighbors of       C01Y Check
PLAT242_ALERT_2_G Low    'MainMol' Ueq as Compared to Neighbors of       C027 Check
PLAT242_ALERT_2_G Low    'MainMol' Ueq as Compared to Neighbors of       C02C Check
PLAT242_ALERT_2_G Low    'MainMol' Ueq as Compared to Neighbors of       C02H Check
PLAT242_ALERT_2_G Low    'MainMol' Ueq as Compared to Neighbors of       C02J Check
PLAT303_ALERT_2_G Full Occupancy Atom H00E      with # Connections       2.00 Check
PLAT720_ALERT_4_G Number of Unusual/Non-Standard Labels ..........        160 Note
              Ag01    Ag02    Ag03    O004    O005    O006    O007    O008
              O009    O00A    F00B    O00C    H00C    F00D    O00E    H00E
              O00F    O00G    H00G    F00H    O00I    H00I    F00J    F00K
              F00L    F00N    O00O    H00O    N00P    N00Q    N00R    N00S
```

```
              F00T    N00U    C00V    N00W    N00X    F00Y    F00Z    C010
              H010    C011    H01A    H01B    F012    F013    F014    C015
              H015    C016    H016    C017    F018    C019    C01A    H01C
              H01D    C01B    C01C    H01E    C01D    H01F    F01E    C01F
              C01G    H01G    F01H    C01I    H01I    C01J    C01K    H01K
              C01L    C01M    H01M    C01N    H01H    H01J    H01L    C01O
              H01N    H01O    H01P    C01P    H01Q    H01R    H01S    C01Q
              H01T    H01U    H01V    C01R    H01W    C01S    H01X    C01T
              H01Y    Ha      C01U    H01Z    Hb      Hc      C01V    H01
              C01W    C01X    C01Y    C01Z    C020    H020    C021    C022
              C023    H02A    H02B    H02C    C024    C025    H02D    H02E
              H02F    C026    H02G    H02H    H02I    C027    C028    C029
              H02J    H02K    C02A    H02L    C02B    H02M    H02N    H02O
              C02C    F02D    C02E    H02P    H02Q    H02R    C02F    H02S
              H02T    C02G    H02U    C02H    F02I    C02J    C02K    H02V
              H02W    H02X    C02L    H02Y    C02M    H02Z    Hd      He
PLAT791_ALERT_4_G Model has Chirality at C010       (Sohncke SpGr)          S Verify
PLAT791_ALERT_4_G Model has Chirality at C015       (Sohncke SpGr)          R Verify
PLAT791_ALERT_4_G Model has Chirality at C016       (Sohncke SpGr)          S Verify
PLAT791_ALERT_4_G Model has Chirality at C017       (Sohncke SpGr)          R Verify
PLAT791_ALERT_4_G Model has Chirality at C01C       (Sohncke SpGr)          S Verify
PLAT791_ALERT_4_G Model has Chirality at C01D       (Sohncke SpGr)          S Verify
PLAT791_ALERT_4_G Model has Chirality at C01G       (Sohncke SpGr)          R Verify
PLAT791_ALERT_4_G Model has Chirality at C01I       (Sohncke SpGr)          R Verify
PLAT791_ALERT_4_G Model has Chirality at C01J       (Sohncke SpGr)          S Verify
PLAT791_ALERT_4_G Model has Chirality at C01M       (Sohncke SpGr)          R Verify
PLAT791_ALERT_4_G Model has Chirality at C01R       (Sohncke SpGr)          S Verify
PLAT791_ALERT_4_G Model has Chirality at C01S       (Sohncke SpGr)          R Verify
PLAT791_ALERT_4_G Model has Chirality at C01V       (Sohncke SpGr)          R Verify
PLAT791_ALERT_4_G Model has Chirality at C01Z       (Sohncke SpGr)          R Verify
PLAT791_ALERT_4_G Model has Chirality at C020       (Sohncke SpGr)          R Verify
PLAT791_ALERT_4_G Model has Chirality at C021       (Sohncke SpGr)          R Verify
PLAT791_ALERT_4_G Model has Chirality at C02A       (Sohncke SpGr)          R Verify
PLAT791_ALERT_4_G Model has Chirality at C02G       (Sohncke SpGr)          R Verify
PLAT883_ALERT_1_G Absent Datum for _atom_sites_solution_primary ..     Please Do !
PLAT910_ALERT_3_G Missing FCF Reflection(s) Below Theta(Min)[Deg]=       3.67 Note
               0  0  1,
PLAT912_ALERT_4_G Missing # of FCF Reflections Above STh/L=  0.600        229 Note
PLAT941_ALERT_3_G Average HKL Measurement Multiplicity ..........         3.6 Low
PLAT969_ALERT_5_G The 'Henn et al.' R-Factor-gap value ..........       2.046 Note
              Predicted wR2: Based on SigI**2  5.83 or SHELX Weight 10.80
PLAT978_ALERT_2_G Number C-C Bonds with Positive Residual Density.          0 Info
```

---

0 **ALERT level A** = Most likely a serious problem - resolve or explain
1 **ALERT level B** = A potentially serious problem, consider carefully
8 **ALERT level C** = Check. Ensure it is not caused by an omission or oversight
33 **ALERT level G** = General information/check it is not something unexpected

2 ALERT type 1 CIF construction/syntax error, inconsistent or missing data
11 ALERT type 2 Indicator that the structure model may be wrong or deficient
5 ALERT type 3 Indicator that the structure quality may be low
22 ALERT type 4 Improvement, methodology, query or suggestion
2 ALERT type 5 Informative message, check

---

## Validation response form

Please find below a validation response form (VRF) that can be filled in and pasted into your CIF.

```
# start Validation Reply Form
_vrf_PLAT042_lcz111
;
PROBLEM: Calc. and Reported MoietyFormula Strings  Differ     Please Check
RESPONSE: ...
;
_vrf_PLAT213_lcz111
;
PROBLEM: Atom F02D            has ADP max/min Ratio .....        3.3 prolat
RESPONSE: ...
;
_vrf_PLAT234_lcz111
;
PROBLEM: Large Hirshfeld Difference F018     --C01Y     .       0.17 Ang.
RESPONSE: ...
;
_vrf_PLAT241_lcz111
;
PROBLEM: High   'MainMol' Ueq as Compared to Neighbors of         O1 Check
RESPONSE: ...
;
_vrf_PLAT342_lcz111
;
PROBLEM: Low Bond Precision on  C-C Bonds ..............      0.013 Ang.
RESPONSE: ...
;
_vrf_PLAT911_lcz111
;
PROBLEM: Missing FCF Refl Between Thmin & STh/L=    0.600         22 Report
RESPONSE: ...
;
_vrf_PLAT915_lcz111
;
PROBLEM: No Flack x Check Done: Low Friedel Pair Coverage         71 %
RESPONSE: ...
;
# end Validation Reply Form
```

---

It is advisable to attempt to resolve as many as possible of the alerts in all categories. Often the minor alerts point to easily fixed oversights, errors and omissions in your CIF or refinement strategy, so attention to these fine details can be worthwhile. It is up to the individual to critically assess their own results and, if necessary, seek expert advice.

---

**PLATON version of 17/09/2025; check.def file version of 17/09/2025**

---

# duplicate check

## No duplication found

---

**Datablock lcz111** - ellipsoid plot

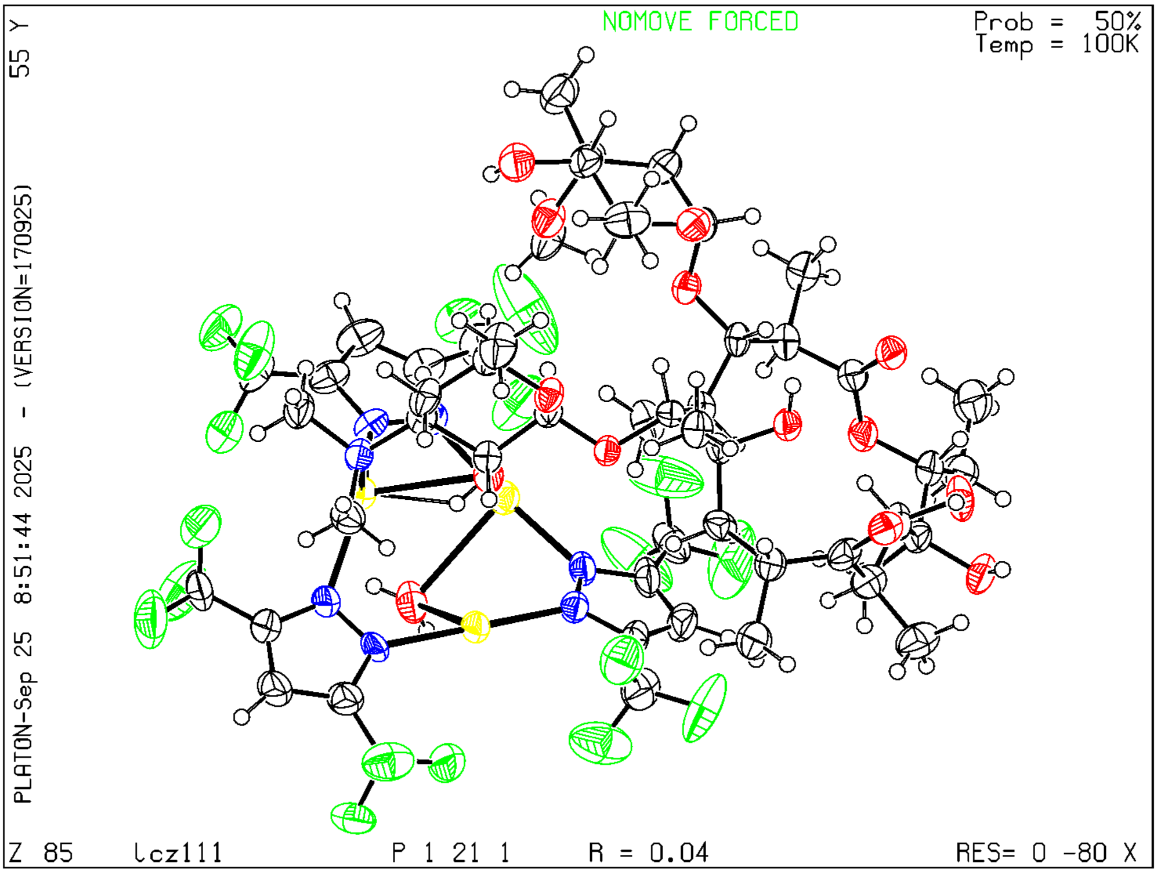

Supplement: Supplementary file 2 — Supporting File 2: anie72462‐sup‐0002‐DataFile.zip. [file ANIE-65-e1218503-s002.zip › supplementary_materials_crystallographic_data submit.zip!/cif_and_checkcif(1-114)/Ag3Pz3_111/Ag3Pz3_111_checkcif.pdf]
